# Characterization of TRKA signaling in acute myeloid leukemia

**DOI:** 10.18632/oncotarget.25723

**Published:** 2018-07-10

**Authors:** Shelley M. Herbrich, Sankaranarayanan Kannan, Riitta M. Nolo, Marisa Hornbaker, Joya Chandra, Patrick A. Zweidler-McKay

**Affiliations:** ^1^ Department of Leukemia, University of Texas M.D. Anderson Cancer Center, Houston, Texas, United States of America; ^2^ Department of Pediatrics, University of Texas M.D. Anderson Cancer Center, Houston, Texas, United States of America; ^3^ Immunogen, Inc., Waltham, Massachuessettes, United States of America

**Keywords:** Acute Myeloid Leukemia (AML), NGF/TRKA signaling, leukemogenesis

## Abstract

Tropomyosin-related kinase A (TRKA) translocations have oncogenic potential and have been found in rare cases of solid tumors. Accumulating evidence indicates that TRKA and its ligand, nerve growth factor (NGF), may play a role in normal hematopoiesis and may be deregulated in leukemogenesis. Here, we report a comprehensive evaluation of TRKA signaling in normal and leukemic cells. TRKA expression is highest in common myeloid progenitors and is overexpressed in core binding factor and megakaryocytic leukemias, especially Down syndrome-related AML. Importantly, NGF can rescue GM-CSF dependent TF-1 AML cells, but does not drive proliferation in other TRKA-expressing lines. Although TRKA expression is heterogeneous between and within AML samples, NGF stimulation broadly induces ERK signaling, demonstrating the functional ability of AML cells to respond to NGF/TRKA signaling. However, neither shRNA knockdown nor pharmacologic inhibition have significant anti-proliferative effects on human AML cells *in vitro* and *in vivo*. Thus, despite functional NGF/TRKA signaling, the importance of TRKA in AML remains unclear.

## INTRODUCTION

Acute myeloid leukemia (AML) is a rapid and aggressive malignancy of the blood in which aberrant myeloid blast cells commandeer the host bone marrow and outcompete normal blood production. Approximately 75% of those diagnosed with acute myeloid leukemia will die within 5 years; a statistic that has remained relatively unchanged for the past decade. The current regiments of high-dose chemotherapy and allogeneic stem cell transplant alone are not sufficient and new strategies for treating myeloid malignancies are vital [1]. One such strategy involves pursuing therapeutics specifically targeting mutated proteins or abnormal pathway activity in the hopes of improving efficacy and reducing toxicity [2]. The genomic era has provided an unprecedented appreciation for the array of genetic and epigenetic alterations that contribute to the initiation and progression of acute myeloid leukemia [3]. While now identified, many of these lesions are not currently drugable. There has, however, been success in selectively targeting aberrant kinase signaling, including the AML subsets with FLT3-internal tandem duplications. Thus, there is considerable interest in understanding novel kinase activity that contributes to AML proliferation and survival that may lead to novel avenues of therapy [4].

Tropomyosin receptor kinase A (TRKA) is a membrane-bound, glycosylated receptor tyrosine kinase and the high-affinity binding partner of nerve growth factor (NGF). This protein is a member of the neurotrophic tyrosine kinase receptor (NTKR) family and is encoded by the NTRK1 gene. TRKA has well-established functions in the survival and differentiation of neurons [5–7]. While predominantly studied in the nervous system, TRKA is known to play an important role in repair and survival processes in a variety of other tissues where NGF can act as a local growth factor [8]. In fact, TRKA expression has been identified in normal hematopoietic stem and progenitor cells and the NGF/TRKA signaling pathway has been implicated in normal myeloid progenitor cell function, differentiation, and survival [9–13]. Interestingly, NGF is secreted by normal bone marrow stromal cell where it can synergize with stem cell factor (SCF) to support stem/progenitor cell populations within the bone marrow microenvironment [14].

High levels of TRKA expression have also been observed in myeloid malignancies. Kaebisch *et al* first demonstrated almost 20 years ago, in the pre-TCGA era, that 44% of leukemic cells from primary AML patients expressed detectable levels of NTRK1 mRNA. However, none of these cells appeared to express the corresponding ligand, negating an AML-driven autocrine loop [15]. Since then, multiple studies have confirmed this frequency of NTRK1 mRNA expression in independent AML cohorts. While levels of NTRK1 do not track with FAB classification, they do appear to be enriched in some cytogenetic subgroups. In one study, AML1-ETO positive AML samples had significantly higher levels of NTRK1 mRNA when compared to other common subtypes of AML [16].

In human AML cell lines, TRKA expression has been reported in erythroleukemic subtypes and can support proliferation of these cell lines with exogenous NGF in place of the typically required granulocyte-monocyte colony-stimulating factor (GM-CSF) [11]. In CD34+ hematopoietic cells, the induction of the AML1-ETO fusion protein was shown to result in an up-regulation of NTRK1 mRNA and NGF-dependent proliferation [16]. Co-expression of the high-affinity receptor TRKA, and its corresponding ligand in murine hematopoietic cells was also shown to be sufficient for leukemogenesis [15–17]. Furthermore, an AML-derived constitutively active TRKA variant was shown to alter apoptosis and contribute to leukemic transformation in murine 32D cells [18].

TRKA is translocated in an array of solid tumors [19–22] and is a proven oncogene when constitutively activated. Although rare, a TRKA family member, TRKC, has leukemogenic potential in the setting of the ETV6-NTRK3 fusion gene [23]. We hypothesized that, while not mutated or translocated in AML, aberrant upregulation of this tyrosine kinase in a subset of disease may prove to be a contributor to AML cell growth or survival and, thus, a candidate for targeted therapy. Despite available clinical drugs, these have not yet been tested in AML. Here, we have provided a systematic analysis of NGF/TRKA signaling encompassing both normal hematopoietic cell types and major subsets of AML. We have also taken the first look at at the heterogeneity of TRKA expression and signaling at the single-cell level using mass cytometry.

## RESULTS

### NTRK1 mRNA is expressed in normal hematopoiesis and overexpressed in AML

**Figure 1:**
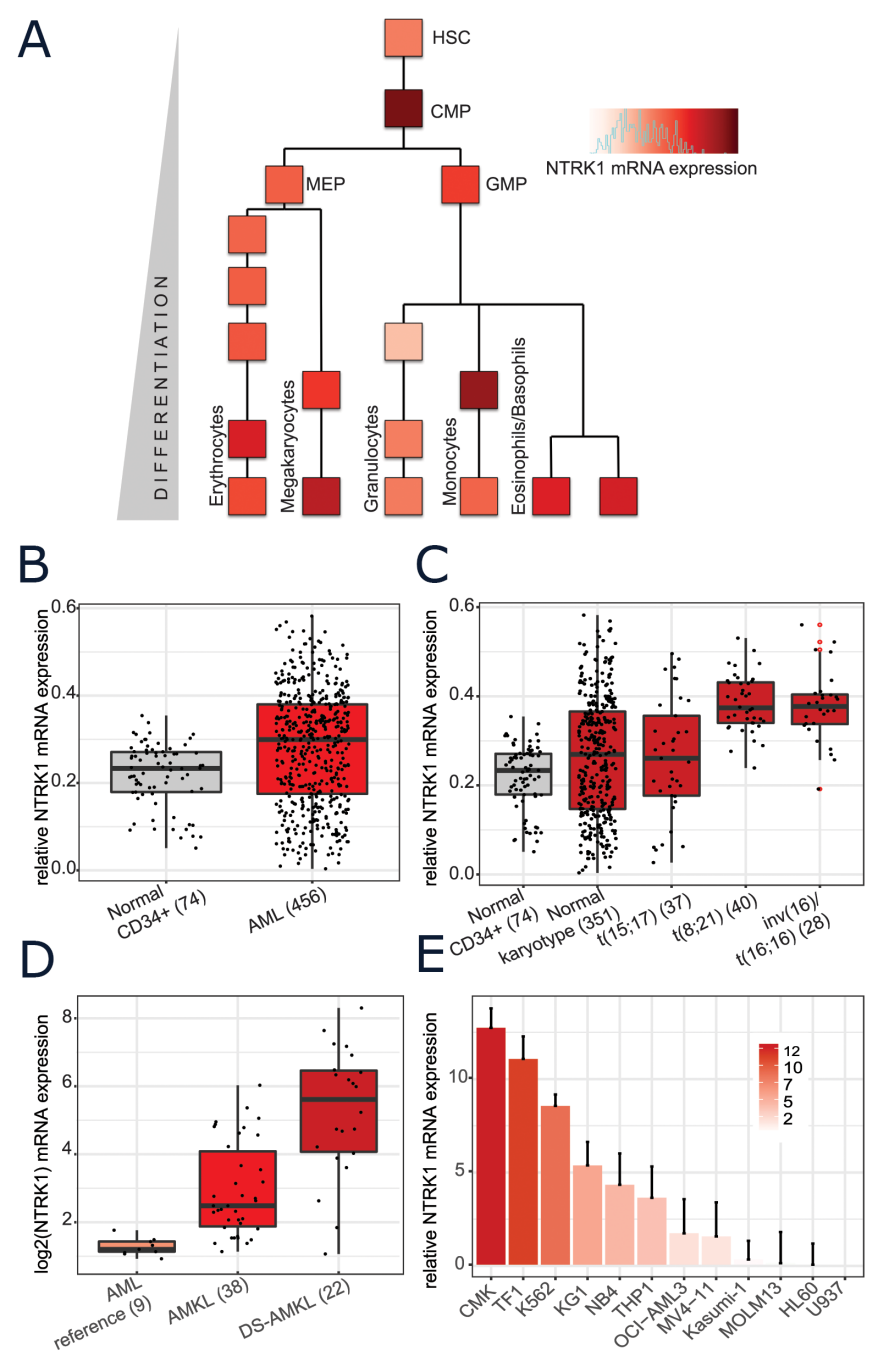
Normal and leukemic human myeloid cells express NTRK1 mRNA **(A)** NTRK1 mRNA expression in the context of the myeloid compartment extracted from the Novershtern *et al* gene expression dataset [24]. Each block represents the average of approximately 6 cell cultures. Cell populations are described in the original manuscript. **(B)** Expression levels of NTRK1 mRNA from normal CD34+ bone marrow cells (gray) and blasts from AML patient samples (red) profiled by the Microarray Initiative in Leukemia Expression (MILE) study. **(C)** Expression of NTRK1 from the MILE database further stratified by AML subtype. The highest levels of expression are observed in in t(8;21) and inv(16)/t(16;16) translocated tumors. **(D)** NTRK1 mRNA collected as part of Bourquin *et al* microarray gene expression study where M4/M5 leukemia was used as a reference for AMKL in both normal and Down syndrome individuals. **(E)** Quantitative RT-PCR (qRT-PCR) analysis of NTRK1 mRNA expression of a panel of 12 AML cell lines representing a variety of AML subsets. Results are represented as a relative fold-change after normalizing with GAPDH mRNA. Each value corresponds to the mean ± SEM of three independent experiments. TRKA mRNA is detectable in 92% (11/12) of cell lines with a range of expression approximately 15-fold. HSC= hematopoietic stem cell, CMP= common myeloid progenitor, MEP= megakaryocytic erythroid progenitor, GMP= granulocytic myeloid progenitor. AMKL=acute megakaryoblastic leukemia, DS= Down syndrome.

The dataset generated by Novershtern *et al* provides genome-wide expression profiling at various stages throughout normal human hematopoiesis (with an average of 6 replicates per stage) [24]. Based on this representation of normal myeloid development, the highest levels of NTRK1 appear in the common myeloid progenitors (CMP) and early monocytes. Appreciable levels of NTRK1 mRNA are also seen in normal erythrocytes, mature megakaryocytes, basophils, and eosinophils (Figure 1A). Several previous studies have observed NTRK1 expression at the transcript level in subsets of human AML samples [13, 25–29]. We mined large, publically available datasets to systematically examine NTRK1 mRNA expression in AML patient samples. Results from the Microarray Innovations in Leukemia (MILE) study allowed for the comparison of various AML subtypes to normal hematopoietic stem cells [30]. NTRK1 was significantly higher in the combined group of 456 AML patients when compared to normal CD34+ bone marrow cells (p < 2.2e-16) (Figure 1B). Partitioning AML samples by cytogenetic subtypes, the highest expression of NTRK1 mRNA was seen in those with t(8,21) and inv(16)/t(16;16), both core binding factor leukemias (Figure 1C). To investigate acute megakaryoblastic leukemia (AMKL), a subset not represented in MILE, we utilized the data from Bourquin *et al* originally generated to define molecular phenotypes of AMKL in both normal and Down syndrome individuals [31]. When compared to AML controls, NTRK1 expression is significantly higher in AMKL and even higher in Down syndrome patients with AMKL (p=0.026, p=0.041) (Figure 1D). We assembled a representative panel of 12 human AML cell lines to confirm NTRK1 mRNA expression *in vitro*. Of the 12 human AML cell lines, NTRK1 transcripts were present at detectable levels in 11 (92%) (Figure 1E). The highest expression was observed in the megakaryoblastic, erythroleukemic, and chronic myeloid leukemia in blast crisis lines (CMK, TF-1, and K562, respectively). Interestingly, the cell line with the t(8;21) translocation, Kasumi-1, expressed only low levels of NTRK1. It is also worth noting that these cell lines did not express the NGF ligand.

### A subset of normal and leukemic subtypes express functional, surface TRKA protein

**Figure 2:**
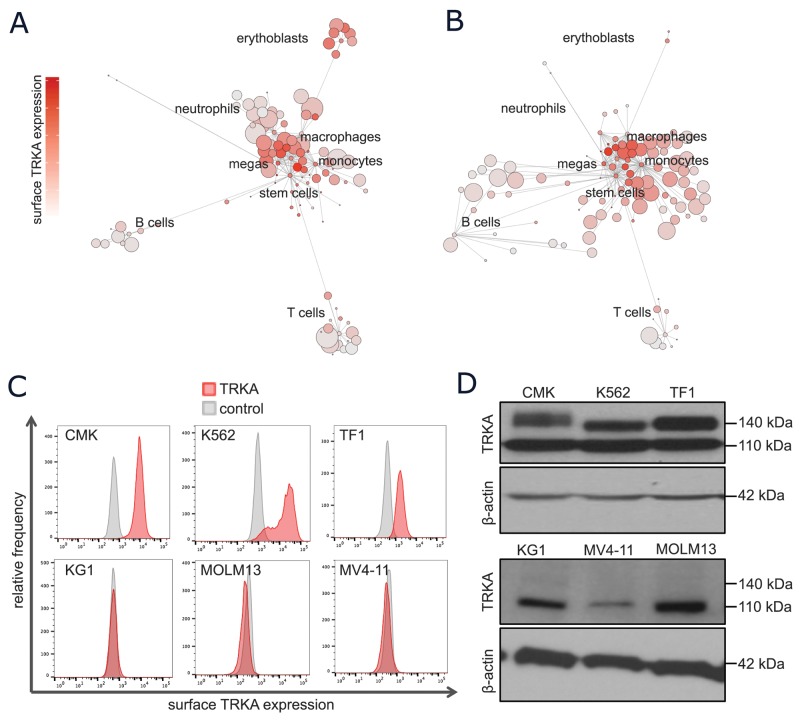
A subset of normal and leukemic myeloid cells express surface TRKA protein **(A-B)** Scaffold visual summary of single cell mass cytometry data depicting total surface TRKA expression in the context of normal hematopoiesis (A) and an example AML sample (B). **(C)** Cells were stained with anti-TRKA-phycoerythrin (PE) (red) and an isotype control mAb (grey). The cell lines that expressed evident surface TRKA, CMK, K562, and TF-1, are displayed along with 3 cell lines that express NTRK1 mRNA but no surface TRKA protein. **(D)** A western blot analysis of TRKA protein expression was performed on cell lysates prepared from the same cells as the previous flow experiment using a rabbit anti-TRKA antibody with a β-actin loading control. The anti-TRKA antibody identifies both the immature (110 kDa) and mature glycosylated (typically 140 kDa) form of the TRKA protein.

The NTRK1 mRNA expression patterns in normal and leukemic cells were confirmed at the single-cell, protein level using mass cytometry. CyTOF was performed on PBMCs from healthy and diseased human bone marrow aspirates and a representative SCAFFoLD map of each are displayed in Figures 2A-2B, respectively. The antibody panel was designed to discriminate various normal, hematopoietic subsets including myeloid stem and progenitor cells, monocytes, granulocytes, megakaryocytes, erythrocytes, B cells, and T cells. This map is colored by surface TRKA protein to allow for the identification of cell types with high TRKA expression. In normal hematopoietic cell samples, TRKA was again highest in early myeloid progenitors, followed by megakaryocytes and erythrocytes (Figure 2A). In the AML SCAFFoLD map, there is an emergence of abnormal cell clusters, representing the leukemia, which express moderate to high levels of TRKA on the cell surface (Figure 2B).

The full-length isoform of TRKA is synthesized as a 110-kDa glycoprotein [32]. Post-translational glycosylation results in the mature 140-kDa form that is capable of translocating to the cell surface where it is able to bind extracellular NGF [33]. To determine which AML cell lines expressed the fully matured, transmembrane TRKA protein, all cells were probed for surface TRKA expression and measured by flow cytometry. The 3 cell lines with the highest mRNA expression, CMK, K562, and TF-1 cells, were the only with clear shifts in TRKA fluorescence when compared to the IgG isotype control (Figure 2C). All remaining cells expressed no detectable surface TRKA protein by flow cytometry (Supplementary Figure 1). Intra- and extra-cellular TRKA protein expression was further validated by Western immunobloting. All cell lines with the exception of HL60 and U937 expressed the immature 110-kDa form of TRKA (Supplementary Figure 2). Consistent with the flow cytometry results, only CMK, K562, and TF-1 cells expressed a glycosylated form of TRKA (Figure 2D).

### NGF stimulates growth and survival in a cytokine dependent cell line

**Figure 3:**
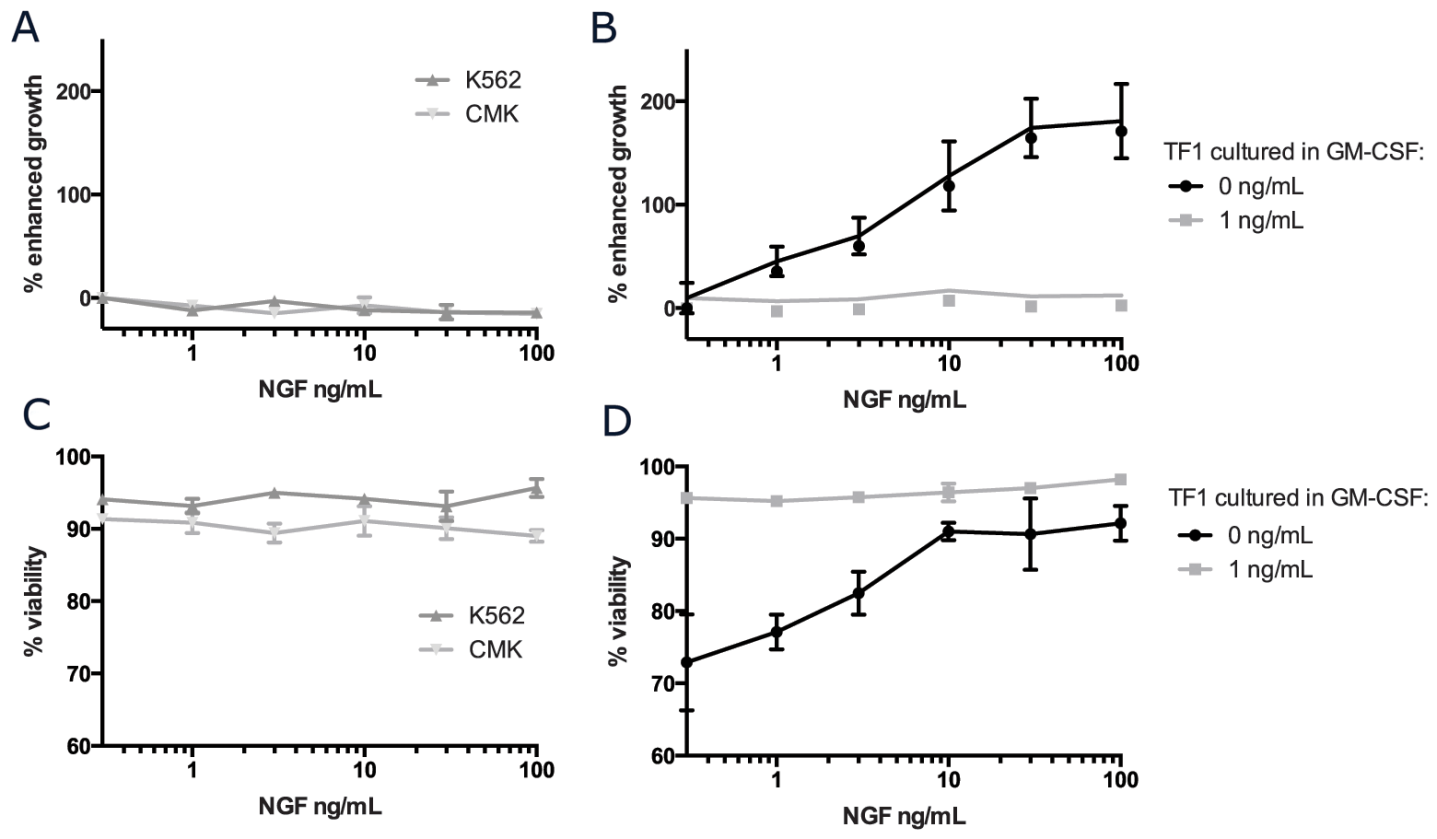
NGF/TRKA signaling can support growth and viability in the cytokine dependent cell line, TF-1 CMK, K562, and TF-1 cells were plated at a density of 5x10^4^ cells/mL and cultured in standard conditions with increasing concentrations of NGF for 5 days. Additionally, for TF-1 cells that are traditionally cultured with supplemental GM-CSF, cells were cultured with either 1 or 0 ng/mL of GM-CSF. **(A-B)** Absolute cell numbers and **(C-D)** viability were measured using Trypan Blue staining, assessed with ViCell, and normalized to cells grown in identical culture conditions without any addition of NGF.

To determine whether functional NGF/TRKA signaling contributes to cell growth or survival, cells with surface TRKA were cultured in increasing concentrations of NGF and outputs of growth and viability were measured and compared to standard culture conditions without any supplemental NGF (Figure 3). The addition of NGF to the culture media did not result in accelerated growth or improved survival in either K562 or CMK cells (Figure 3A, 3C). We were, however, able to confirm that NGF could induce proliferation and sustain survival in the cytokine dependent cell line, TF-1 (Figure 3B, 3D) [11]. In contrast to the previously reported results, 100 ng/mL of NGF was only 20% as effective as 2 ng/mL of GM-CSF (the standard culture condition for TF-1 cells) in terms of supporting cell growth. Additionally, while NGF alone was able to support the growth and survival of TF-1 cells in a dose dependent manor, this effect was abrogated in the presence of even trace amounts (<1 ng/mL) of GM-CSF. There was no additive effect on growth or proliferation with the combination of NGF and GM-CSF in TF-1 cells. We confirmed that TF-1 cells have a much stronger affinity for downstream signaling in response to GM-CSF when compared to NGF (Supplementary Figure 3).

### Mature TRKA in AML is capable of signaling upon NGF stimulation

**Figure 4:**
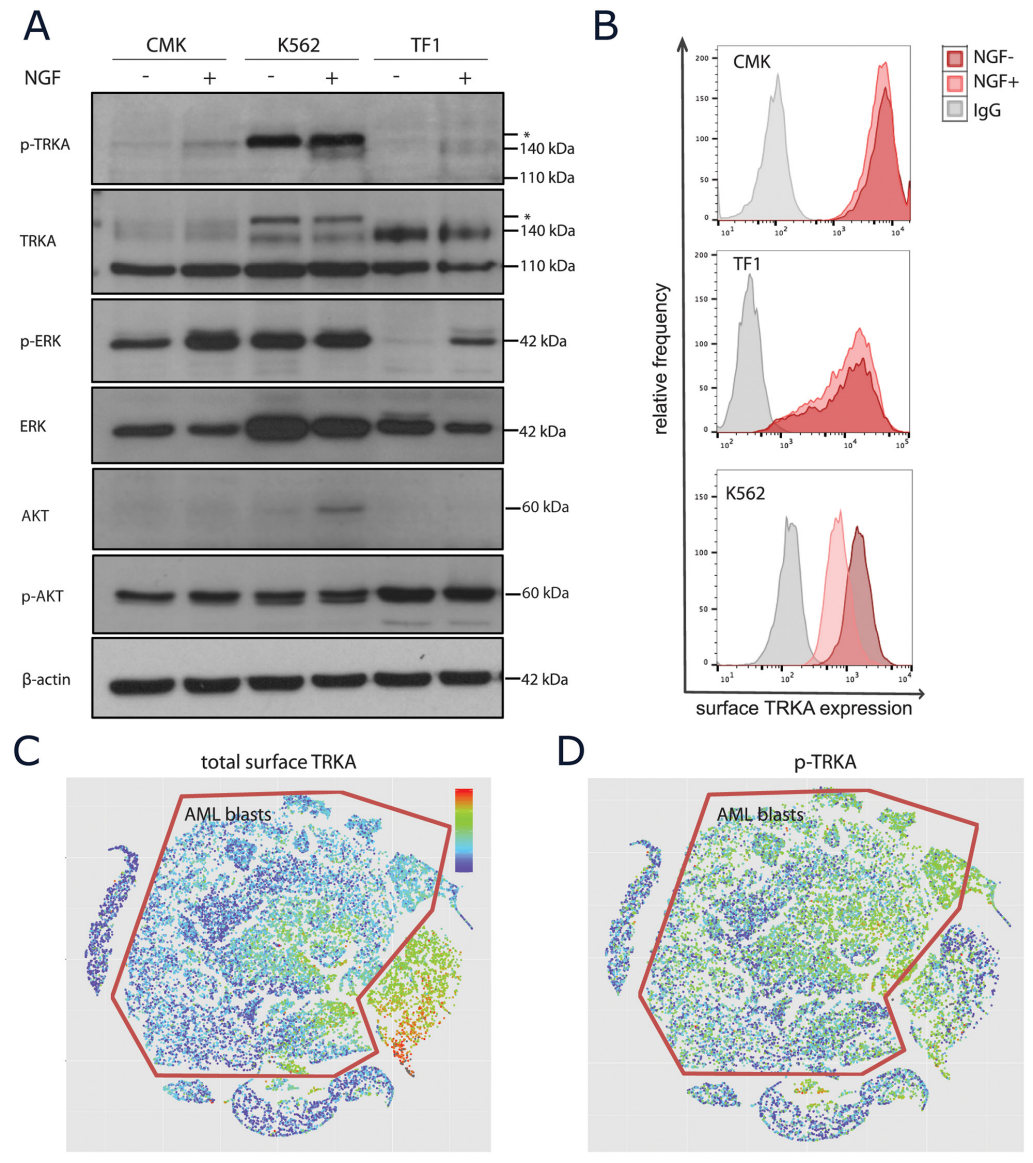
TRKA signaling upon NGF stimulation **(A)** Western blot analysis of unstimulated or NGF-stimulated (100 ng/mL for 15 minutes at 37°C) extracts from CMK, K562, and TF-1 cells using anti-p-TRKA, ant-TRKA, anti-p-ERK, anti-ERK, anti-p-AKT, and anti-AKT antibodies with a β-actin loading control. The p-TRKA antibody is reported to be cross reactive with the ABL protein, known to be constitutively expressed in K562 cells. Asterisks indicate additional protein bands recognized by the anti-p-TRKA antibody. **(B)** Surface flow cytometry of the TRKA protein to determine functional signaling upon NGF stimulation. **(C-D)** Pooled normal BM and 4 AML patient samples stimulated with NGF were projected onto the same ViSNE map. Blast cells were identified using cell surface protein markers and are outlined in red. While there appears to be moderate baseline expression of surface TRKA (C), there is p-TRKA activity throughout the blast population upon NGF stimulation (D).

To determine whether or not the mature TRKA protein in these AML cells was capable of functional signaling, cells were stimulated with NGF followed by measurement of both phospho-protein expression and receptor turnover. We chose to use the ERK1/2 and AKT signaling pathways as surrogate measurements of activity as these are the most widely reported downstream targets of TRKA in the literature [34]. There was evidence of auto-phosphorylation of TRKA upon ligand stimulation in all 3 cell lines with surface TRKA (Figure 4A). Only TF-1 cells have a clear on/off pattern of p-ERK1/2 signaling in the presence of NGF. Both CMKs and K562s have constitutive p-ERK signaling due to other receptor tyrosine kinase alterations, however, there was still evidence of a moderate increase in p-ERK upon the addition of NGF to the CMK cells. The K562 cell line was the only model to use the AKT signaling pathway in response to NGF. Due to the weak p-TRKA antibody appearance by western, we confirmed that the downstream signaling upon NGF stimulation was, in fact, dependent on the TRKA receptor using a TRKA shRNA in the TF1 cell line (Supplementary Figure 4). In neurons, TRKA must undergo endocytosis for functional downstream signaling [35]. Thus, we next examined the levels of surface TRKA protein before and after 15 minutes of NGF stimulation. Again, only TF-1 cells showed evidence of signaling endocytosis and/or receptor recycling upon ligand stimulation (Figure 4B). This suggests that TRKA internalization may be necessary for downstream signaling through ERK and that expressing surface TRKA does not ensure signaling through this pathway in the presence of ligand.

As patient samples are more heterogeneous than cell lines, we also measured TRKA signaling in one pooled normal bone marrow and four AML bone marrow biopsy samples at the single-cell resolution using CyTOF. All five samples were simultaneously visualized using ViSNE [36]. The AML blasts expressed moderate levels of surface TRKA when compared to the normal immature myeloid cells (Figure 4C). However, upon NGF stimulation, we detected widespread p-TRKA activity across these blast cells (Figure 4D).

### TRKA signaling is dispensable for AML cell growth and survival

**Figure 5:**
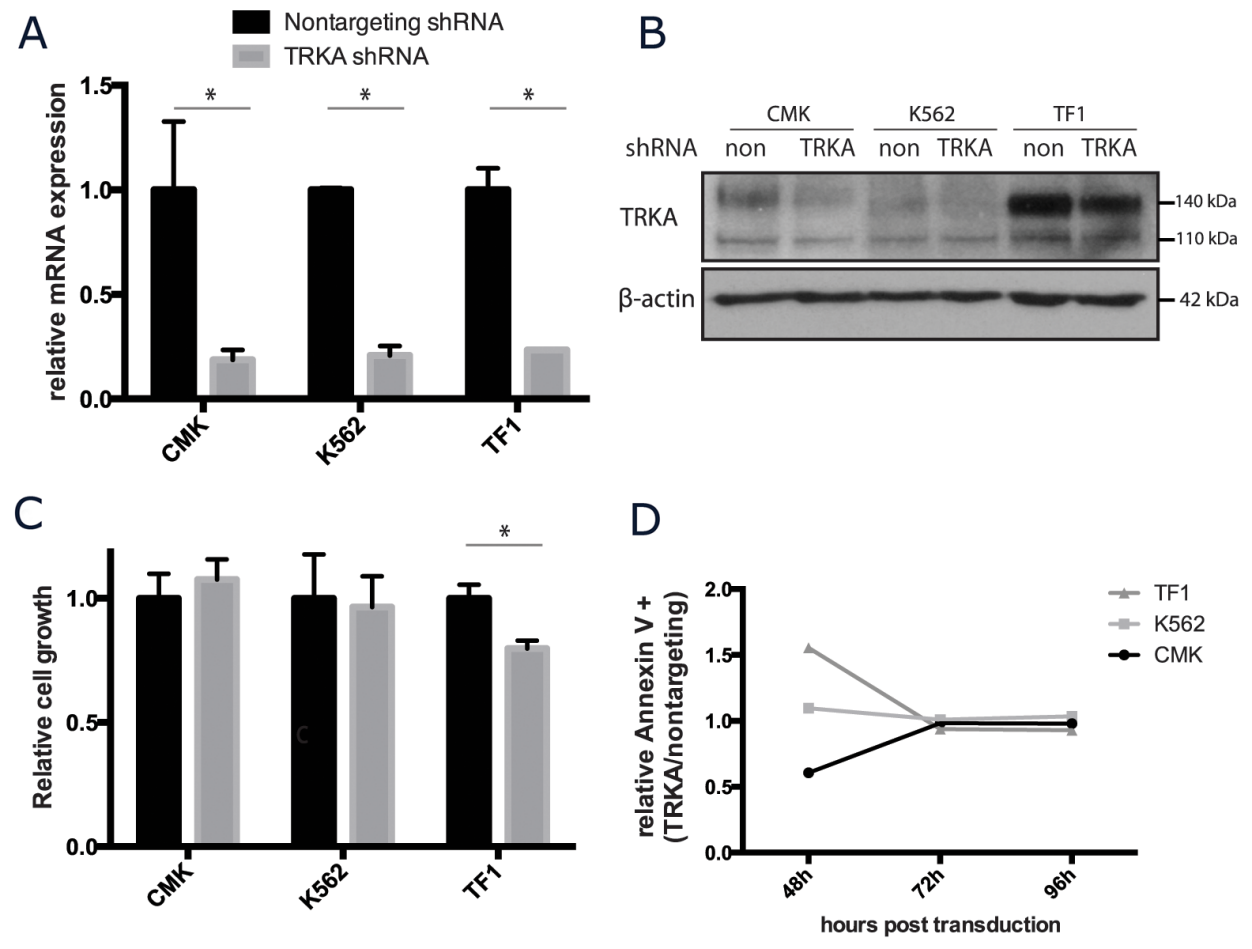
TRKA is not essential for the growth or survival of AML cell lines *in vitro* CMK, K562, and TF-1 cells were stably transfected with shRNA against TRKA or a scrambled control (ctrl) and monitored for changes in growth and viability. **(A)** qRT-PCR analysis of the NTRK1 mRNA levels in the transduced cell lines 7 days after transduction. Values were normalized to GAPDH and results are displayed as the mean mRNA (+s.d.) relative to the cells transfected with the control vector. There is a consistent knockdown of approximately 70% at the mRNA level across the 3 lines. **(B)** Western blot of total TRKA protein 7 days after transduction with either a nontargeting or TRKA shRNA. **(C)** 48 hours after transduction, cells were plated at a density of 5×10^4^ cells/mL and cultured for 5 days. Cell growth and viability were stained with Trypan Blue and assessed using the ViCell. **(D)** Apoptosis was tracked in both control and knockdown cells using Annexin V staining via flow cytometry.

While most cells do not appear to benefit from supplemental NGF, there are small amounts of NGF in the fetal calf serum used in standard culture media. To determine the essentiality of NGF/TRKA signaling in AML, CMK, K562, and TF-1 cells were stably transduced with a short hairpin RNA (shRNA) targeted to TRKA and a scrambled shRNA control. The silencing shRNA significantly reduced NTRK1 mRNA gene expression by approximately 80% for each cell line when compared to the expression in the scrambled control (Figure 5A). By protein quantification, the shRNA construct effectively reduced total TRKA protein (Figure 5B). The reduced protein expression trended towards being more effective in the mature protein (140 kDa) than the immature protein (110 kDa) (mean reduction of 57% and 36%, respectively) (Supplementary Figure 5). Cells transduced with either construct were cultured in identical conditions and monitored for growth and survival over the course of one week. Viability of all cell lines at the end of 5 days was unaffected by the reduction of TRKA expression. CMK and K562 cells grew at the same rate regardless of the construct. TRKA knockdown did significantly (p<0.05) reduce the total number of TF-1 cells (Figure 5C). This may be attributable to the fact that TF-1 cells transduced with the TRKA shRNA had a rate of apoptosis 1.5 times that cells transduced with the scrambled control vector 48 hours after transduction. However, this difference in cell death was transient (Figure 5D).

### Pharmacological inhibition of NGF/TRKA signaling is not lethal as a single agent in AML

**Figure 6:**
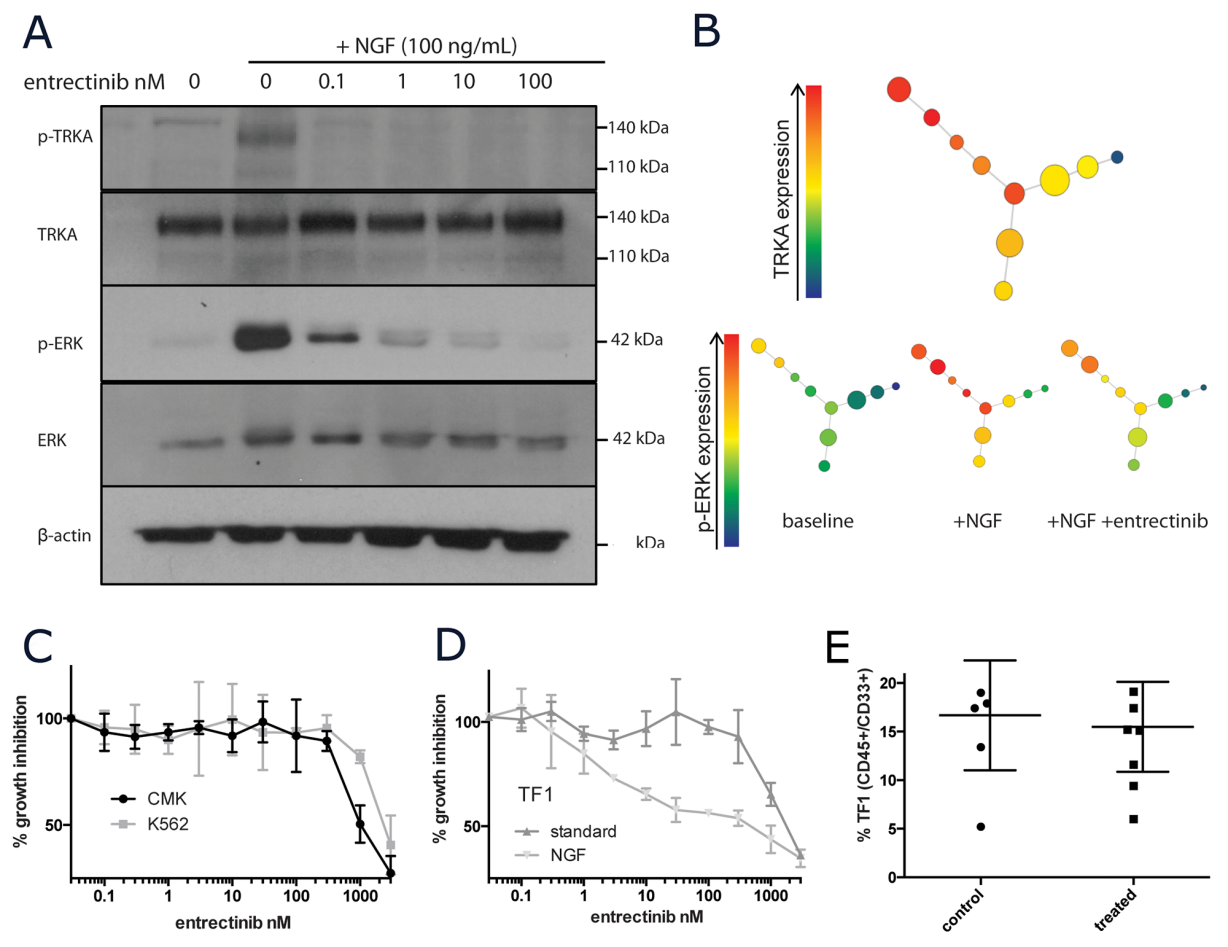
Pharmacological inhibition of TRKA *in vitro* and *in vivo* **(A)** Western blot of NGF/TRKA signaling inhibition with entrectinib. TF-1 cells were incubated with varying concentrations of entrectinib (0-100nM) for 1 hour prior to 15 minutes of NGF (100ng/mL) stimulation. **(B)** SPADE visual summary of CyTOF single cell protein data for TF-1 cells treated with NGF (100ng/mL for 15 minutes) and entrectinib (300nM for 2 hours prior to NGF stimulation). **(C)** Viable cell numbers of CMK, K562, and **(D)** TF-1 (under different culture conditions) were recorded after 72 of incubation with increasing doses of entrectinib (0.1-3000nM) and normalized to their corresponding untreated controls. **(E)** Summary of tumor burden in mice engrafted with TF-1 leukemia after 3 weeks of entrectinib therapy.

Entrectinib is a clinically active small molecule inhibitor of the TRK family of receptors currently in Phase I/II clinical trials in patients with solid tumors harboring TRK fusion proteins [37]. While TRKA genetic alterations are exceedingly rare in AML, we have shown that some subsets of AML express aberrantly high levels of TRKA. We utilized entrectinib as a tool compound to pharmacologically inhibit TRKA *in vitro* and *in vivo*. Entrectinib was able to inhibit TRKA signaling in response to NGF stimulation (100ug/mL for 15 minutes) at low doses (IC50 <10nM). Inhibition of p-TRKA (as detectable by western blotting) was seen as low as 0.1nM (Figure 6A). However, p-ERK levels did not return to baseline until treatment with >100nM of drug (Figure 6A). With the single-cell resolution of CyTOF, we were able to confirm that cells with the highest levels of TRKA protein were those that signal through p-ERK upon NGF stimulation. While largely dampened by incubation with the TRKA inhibitor, there was still detectable p-ERK signaling with NGF even in the presence of 300nM entrectinib (Figure 6B). Growth of CMK and K562 cells was only inhibited at concentrations greater than 1uM (Figure 6C). When supplemented with only NGF, TF-1 cell growth was inhibited with an IC50 of 30nM. This effect was again abrogated by the addition of GM-CSF (Figure 6D).

We developed a TF-1 xenograft model in NSG-SGM3 mice. After allowing TF-1 AML cells one week to engraft, entrectinib was administered twice daily by oral gavage. Mice were monitored until controls showed evidence of severe disease (approximately 4 weeks post injection) and then sacrificed. We measured the detectable amounts of TF-1 cells in the mouse bone marrow by flow cytometry. There was no detectable difference between the control and treatment group (Figure 6E). However, TRKA inhibition appeared to be highly tolerable with no mice from the treatment group presenting with any severe side effects.

## DISCUSSION

The observation of TRKA in AML patient blasts has been around for more than 20 years and yet, the understanding of the biology of TRKA signaling in AML is limited. In the current study, we report a comprehensive characterization of TRKA expression and functionality across a spectrum of AML subtypes.

Consistent with previous reports, we saw a wide range of NTRK1 expression at the mRNA level across patients with various forms of AML [17]. Upon investigation *in vitro*, we observed that NTRK1 mRNA expression was strongly correlated with abundance of the immature 110-kDa form of the TRKA protein. However, only those cell lines with the highest mRNA levels produced the mature 140-kDa protein that is capable of membrane translocation and functional ligand binding in the extracellular space. It is unclear if these immature TRKA proteins play another role in the context of AML. Furthermore, we observed minimal surface clearance of the membrane-bound TRKA protein and downstream signaling upon ligand stimulation in those cell lines with kinase driver mutations. It has been shown elsewhere that CML cells express high levels of TRKA surface protein but do not function upon NGF stimulation. However, when the aberrant BCR/ABL activity is blocked using imatinib, functional TRKA signaling can be restored and induce downstream pathways that ultimately promote cell survival [38]. Thus, other constitutive kinase activity may interfere with normal TRKA signaling. In leukemic cells where TRKA is aberrantly expressed in conjunction with a driver kinase mutation, TRKA may serve as a resistance mechanism and combination targeted therapy studies should be explored. Interestingly, many of the small-molecule inhibitors originally designed to target TRKA or the TRK family of receptors were subsequently found to be extremely potent inhibitors of related receptor tyrosine kinases including FLT3 and JAK2 [39–41]. Conversely, TRKA is a common off-target of JAK and FLT3 inhibitors [42, 43]. Thus, AML patients being treated with such inhibitors may already be benefiting from inadvertent dual therapy.

As AML is an incredibly heterogeneous disease, we anticipated that TRKA expression levels would vary by cytogenetic subtypes. Indeed, patients with t(8;21) and inv(16)/t(16;16) translocations had the highest levels of TRKA expression in both the MILE and TCGA datasets. This is consistent with the findings of Mulloy *et al* who observed that TRKA expression levels are increased as a result of inducing expression of the fusion protein that results from the t(8;21) translocation [16]. Both t(8;21) and inv(16)/t(16;16) translocations involve a member of the core binding factor (CBF) complex and typically results in the overexpression of each component. Research in nociceptive sensory neurons has identified a putative binding site for the CBF transcriptional complex in the minimal enhancer region of TRKA and demonstrated that induction of AML1/RUNX1 is sufficient to induce TRKA expression [44]. In Down syndrome, individuals carry 3 copies of chromosome 21, which results in a 1.5 fold gene dosage increase for all genes on this chromosome, including AML1/RUNX1. These individuals are also at an increased risk for developing acute megakaryoblastic leukemia (AMKL). Thus, we were not surprised to also find high levels of TRKA mRNA in Down syndrome patients with AMKL compared to other cases of AMKL or AML. Together these results indicate that the overexpression of the core binding factor complex could result in the increased TRKA production and could be used a surrogate marker for patients with elevated TRKA.

Chevalier *et al* first demonstrated that NGF alone was capable of supporting growth and proliferation in the cytokine-dependent erythroleukemic cell line, TF-1 [11]. Similar findings were reported in UT-7 cells, a cytokine-dependent cell line derived from a patient with megakaryoblastic leukemia [14]. We show, for the first time, that the reliance on NGF/TRKA signaling in these cells is nullified in the presence of even trace amounts of GM-CSF. This suggests that while NGF/TRKA signaling is capable of supplementing these cells, it is not the preferred signaling pathway and appears to be redundant with signaling induced by GM-CSF. However, these results imply that some cytokine dependent AML cells can survive in neutrally innervated regions absent of hematopoietic cytokines and growth factors.

The fact that there was no difference in tumor burden in the bone marrow of mice treated with the TRKA inhibitor as a single agent versus the vehicle control likely indicated that the majority of cells in the murine bone marrow had sufficient access to GM-CSF or other stimulatory cytokines and were not affected by the inhibition of TRKA. It would be of interest to look, by bioluminescence for example, at the distribution of the AML cells in the mice. This could have potentially identified regions with differential infiltration of TF-1 cells, indicating neural niches where cells are more reliant on NGF. Additionally, rather than attempting TRKA inhibition as a single agent therapeutic, it would be worth exploring the cells that survive standard chemotherapeutic agents or other targeted therapeutics and determining whether or not they rely on NGF/TRKA signaling as a resistance mechanism.

## MATERIALS AND METHODS

### Cell culture

A panel of twelve human AML cell lines; CMK (ACC-392), HL-60 (CCL-240), K562 (CCL-243), Kasumi-1 (CRL-2724), KG1 (CCL-246), MOLM13 (ACC-554), MV4-11 (CLR-9591), NB4 (ACC-207), OCI-AML3 (ACC-582), TF-1 (CLR-2003), THP1 (TIB-202), and U937 (CRL-1593.2) were grown in RPMI-1640 media containing 10% fetal bovine serum, with supplemental pen/strep, Sodium-Pyruvate, HEPES and non-essential amino acids. TF-1 cells were supplemented with 2 ng/mL rGM-CSF (Cat No. 300-03; Peprotech).

### RNA extraction and quantitative real-time PCR (qRT-PCR)

Total RNA from cell lines was extracted using the RNeasy Mini Kit (Cat No. 74104; Qiagen). Reverse transcription was performed with the Sensiscript RT Kit (Cat No. 205111; Qiagen). Real-time PCR primers for TRKA (Hs01021011_m1) and glyceraldehyde-3-phosphate dehydrogenase (GAPDH) (Hs02786624_g1) were purchased from ThermoFisher Scientific. RNA expression was quantified using TaqMan Universal PCR Master Mix (Cat No. 4304437; Applied BioSystems). PCRs were amplified for 60 cycles on a 96-well LightCycler 480 (Roche). All values were normalized to GAPDH expression using the 2^Δ-ΔCt^ method.

### Cell surface flow cytometry

Cell pellets were washed twice with PBS and FC blocked before staining with one or more of the following antibodies; TRKA-phycoerythrin (PE) (Cat No. FAB1751P; R&D Systems);, or mouse IgG1,κ Isotype control (PE) (Cat No. 400114; BioLegend). Cells were incubated according to the individual antibody protocol. Fluorescence was measured using the BD Fortessa.

### Western immunoblotting

Cell lines were stimulated with 100 ng/mL recombinant NGF or 2 ng/mL recombinant GM-CSF and incubated at 37°C for 15 minutes before being lysed using HEPES lysis buffer (1% triton) with phosphatases inhibitory cocktail (SER-TRE Cat No. P2850 and TYR Cat No. P2850; Sigma-Aldrich). Some groups were treated with varying concentrations of the TRK inhibitor, entrectinib, for 2 hours prior to NGF addition. SDS-PAGE, PVDF transfer, and Western immunoblot were all performed using standard techniques (BioRad Manual). Staining antibodies for western immunoblotting included total anti-TRKA (Cat No. ab1445; Abcam) and anti-phosphorylated TRKA-Tyr490 (Cat No. ab37837; Abcam), ERK1/2 (Cat No. AF1576; R&D Systems), p-ERK1/2 Tyr202/Tyr204 (Cat No. AF1018; R&D Systems), and b-actin. Protein bands were quantified using ImageJ for statistical analysis [45].

### Cell viability assays

To assess cell growth and viability, 500ul of each sample condition were collected and counted using the ViCell Cell Counter & Cell Viability Analyzer (Beckman Coulter). Total cell and total viable cell counts were normalized to unstimulated controls.

### Retroviral transduction

The GIPZ human NTRK1 shRNA construct (Clone ID: V2LHS_152388) was purchased from Dharmacon. As a negative control, GIPZ non-silencing lentiviral shRNA control vector (RHS4346) was purchased from Open Biosystems. Both were packaged into lentivirus by the MD Anderson shRNA and ORFeome Core Facility.

For transduction, 1×10^6^ cells were plated in a 24-well tissue culture plate with 250μl of retrovirus and 8 μg/ml of polybrene (Sigma-Aldrich). After centrifugation at 1,000 g for 90 min, the cells were incubated at 37°C in 5% CO_2_ for 6 hours before addition of fresh C10 media. At 2 days, GFP expression was measured by flow cytometry (BD Fortessa).

### Tumorigenicity studies

All NSG-SGM3 mice were housed in the MD Anderson Cancer Center animal facility. All experiments were performed in accordance with guidelines approved by the MD Anderson Cancer Center Institutional Animal Care and Use Committee. TF-1 cells, at a concentration of 1x10^5^/mL, were injected intravenously into previously irradiated NSG-SGM3 mice. After 7 days of engraftment, mice were treated with 60 mg/kg of entrectinib by gavage BID for 3 weeks without toxicity. At the end of treatment, mice were sacrificed and leukemia burden was determined as the percentage of TF-1 cells present in the bone marrow extracted from the femur measured by flow cytometry (BD Fortessa).

### Mass cytometry

Frozen primary bone marrow samples were thawed two days prior to staining to allow for cell recovery. All antibodies were labeled with heavy metals using Maxpar-X8 labeling reagent kits (DVS Sciences) according to manufacturer’s instructions and titrated for optimal concentration determination. For normal and AML bone marrow samples, 1x10^6^ cells were aliquoted into separate FACS tubes. Cells were washed twice with cell staining media (CSM; sterile filtered PBS with 0.5%BSA) and pelleted at 500xg for 5 minutes at 4°C. Cells were blocked with 5 μL Fc-receptor block for 15 minutes at room temperature and then stained in 100 μL of surface antibody master mix (Table in Supplementary Table 1) + CSM for 30 minutes at room temperature. After washing, live cell discrimination was performed by adding 5 μM Cell-ID cisplatin (Cat No. 201064; Fluidigm) to each tube for 30 seconds on a shaker, followed immediately by 3 wash cycles. For intracellular staining, 1 mL of 4°C methanol was added to each pellet and incubated at 4°C overnight. After permeablization, cells were washed with a minimum of 3 mL CSM per tube. Cells were then stained in 100 μL of intracellular antibody master mix (Table in Supplementary Table 1) + CSM for 1 hour at room temperature and washed. For cell discrimination, a metallointercalator working solution (1 mL PBS, 100 μL formaldehyde solution, 0.22 μL iridium metallointercalator (Cat No. 201103A; Fluidigm)) was added to each sample and allowed to incubate for 20 minutes at room temperature. Cells were washed with a minimum of 3 mL CSM, then again with 3 mL ddH2O with 0.1%BSA. Finally, cells were filtered through a 35 μm filter and resuspended in 50 μL ddH2O with 0.1%BSA. The MDACC Flow Cytometry and Cellular Imaging Core Facility prepared the antibody cocktails and acquired data on a CyTOF instrument (DVS Sciences).

### Mass cytometry data analysis

Mass cytometry data files (.fcs) were first filtered using FloJo to remove the normalization beads, debris, doublets, and dead cells. Cleaned data was ported into R (version 3.2.1, The R Foundation for

Statistical Computing) to generate the SCAFFoLD plots using the R package ‘scaffold’ [36, 46–48] and the ViSNE plots using the package ‘Rtsne’ [47]. SPADE figures were generated using the Cytobank software (Cytobank Inc.) [49]. Only surface markers were used as clustering parameters for all 3 algorithms.

### Bioinformatics and statistics

Leukemia data by subsets was collected as part of the MILE study and is publically available through NCBI Gene Expression Omnibus, GEO (GSE13204) [30, 50]. AMKL and DS-AMKL gene expression data was originally generated by Bourquin *et al* and is publically available through NCBI GEO (GSE4119) [31]. All meta-analyses were performed using R [48]. NCBI GEO datasets were obtained using the ‘GEOquery’ R package [51]. Gene expression data was log2 transformed. Statistical analysis of differential mRNA and protein expression was performed using Student’s *t*-test. Analysis of correlation was performed using Spearman’s rank correlation. Statistical differences in experimental data were analyzed using Student’s t-test in GraphPad Prism 6 for MacOSX.

## CONCLUSIONS

This work provides a comprehensive summary of TRKA across a variety of AML subtypes. We demonstrate that while mRNA expression is widespread and signaling is functional in a subset of cells, it is not essential for the growth or survival of the leukemia in the context of the experiments we performed. This study also confirms the observation that in the absence of other cytokines, some leukemia cells can survive in the presence of NGF, introducing the concept that under certain circumstances TRKA inhibition may be beneficial. While TRKA inhibition does not appear to be a singular therapeutic target, the potential remains that the NGF/TRKA pathway may cooperate with oncogenic drivers, and may function as a potential resistance mechanism.

## SUPPLEMENTARY MATERIALS FIGURES AND TABLE



## Abbreviations

TRKA: Tropomyosin receptor kinase A
NTRK1: Neurotrophic tyrosine kinase receptor 1
NGF: Nerve growth factor
GM-CSF: Granulocyte-macrophage colony-stimulating factor
AML: Acute myeloid leukemia
AMKL: Acute megakaryoblastic leukemia
