# Supplementary material for: Characterization of TRKA signaling in acute myeloid leukemia
Source: Oncotarget. 2018 Jul 10;9(53):30092–105. doi: 10.18632/oncotarget.25723 (PMC6059018; doi:10.18632/oncotarget.25723)
Supplement: Supplementary file 1 [file oncotarget-09-30092-s001.pdf]

# Characterization of TRKA signaling in acute myeloid leukemia

## SUPPLEMENTARY MATERIALS

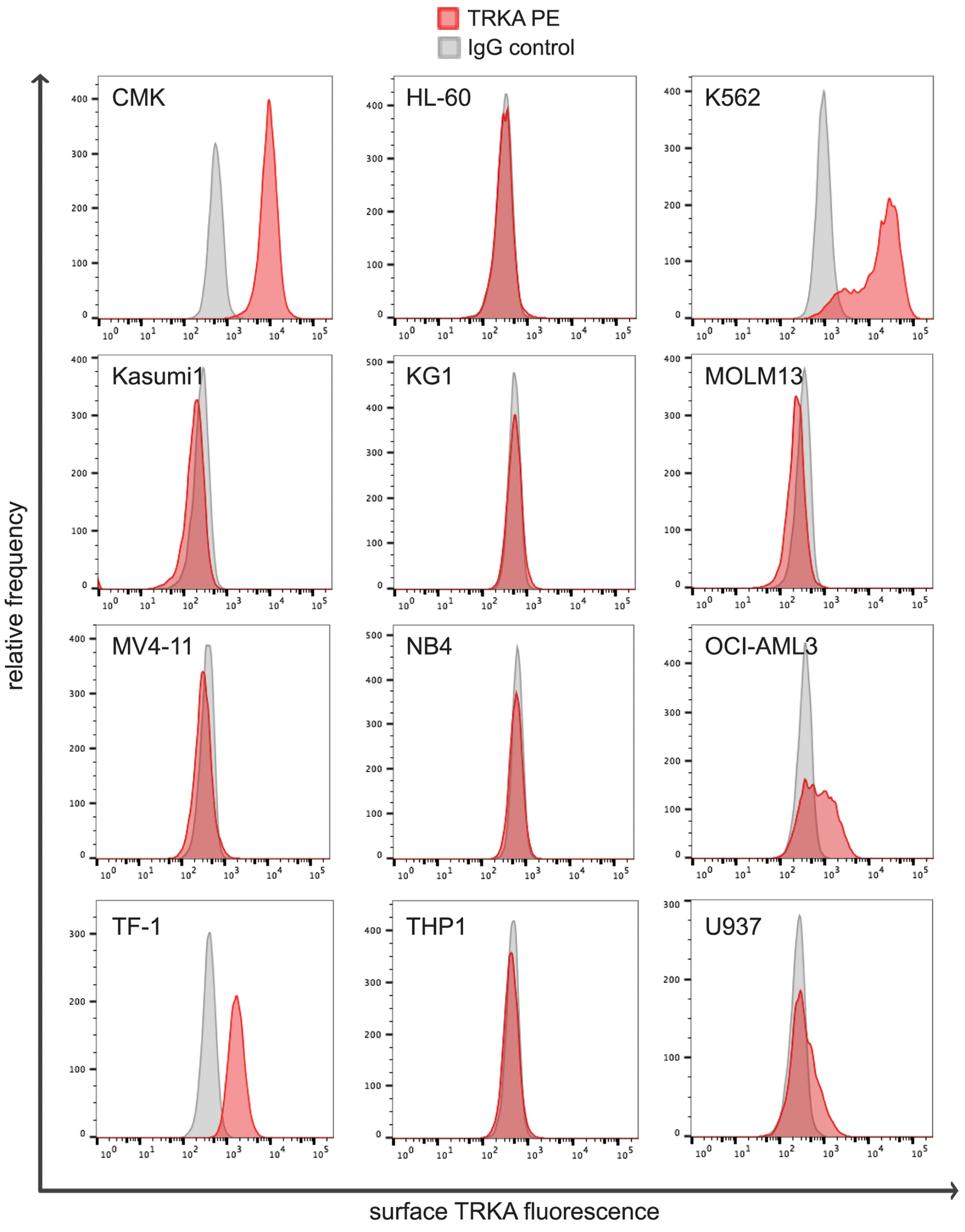


**Supplementary Figure 1: Surface TRKA protein expression across all AML cell lines.** Cells were stained with anti-TRKA-phycoerythrin (PE) (red) and an isotype control mAb (grey). Figures are representative of three replicate experiments with similar results.

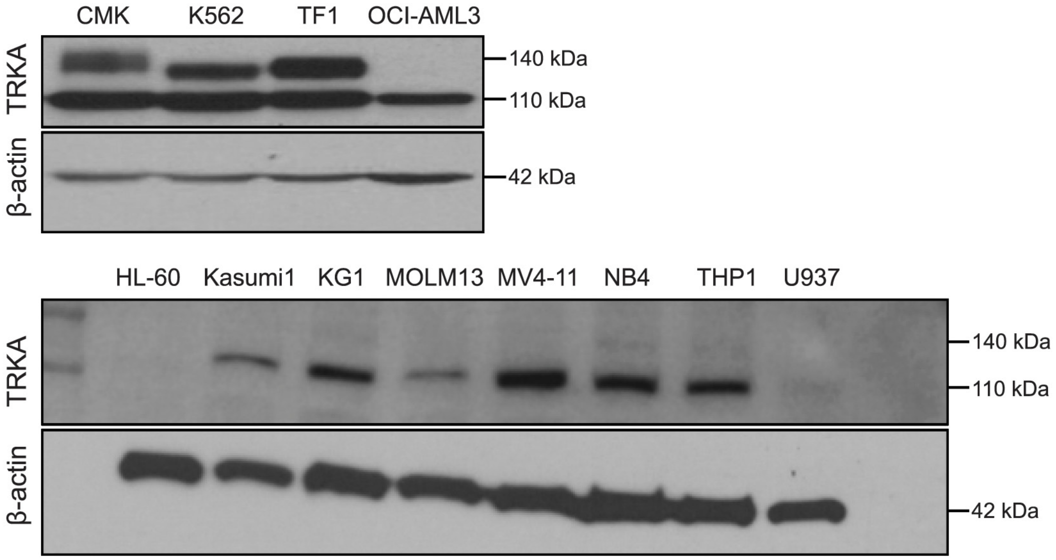


**Supplementary Figure 2: Total TRKA protein expression across all AML cell lines.** Western blot analysis for total TRKA protein expression from the cell lysates prepared from all 12 cell lines, run on 2 8% gels, using a rabbit anti-TRKA antibody with a β-actin loading control. The anti-TRKA antibody identifies both the immature (110 kDa) and glycosylated (typically 140 kDa) form of the TRKA protein.

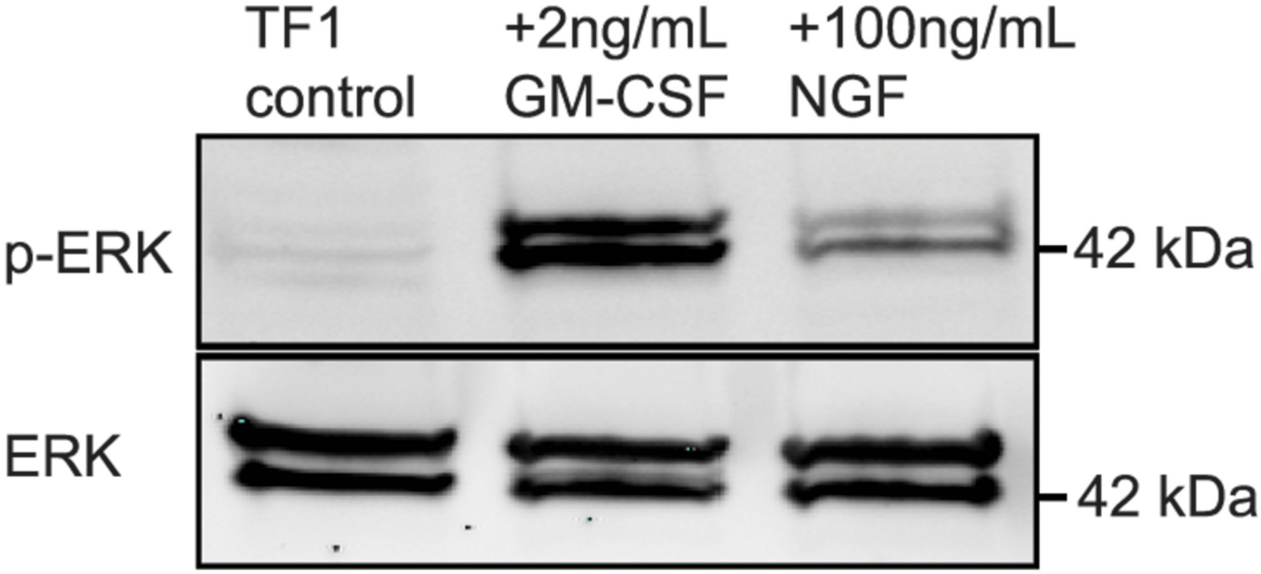


**Supplementary Figure 3: NGF/TRKA signaling with NGF and GM-CSF.** Western blot analysis for phosphor and total ERK in TF-1 cells after stimulation with either 2ng/mL GM-CSF or 100 ng/mL NGF for 15 minutes.

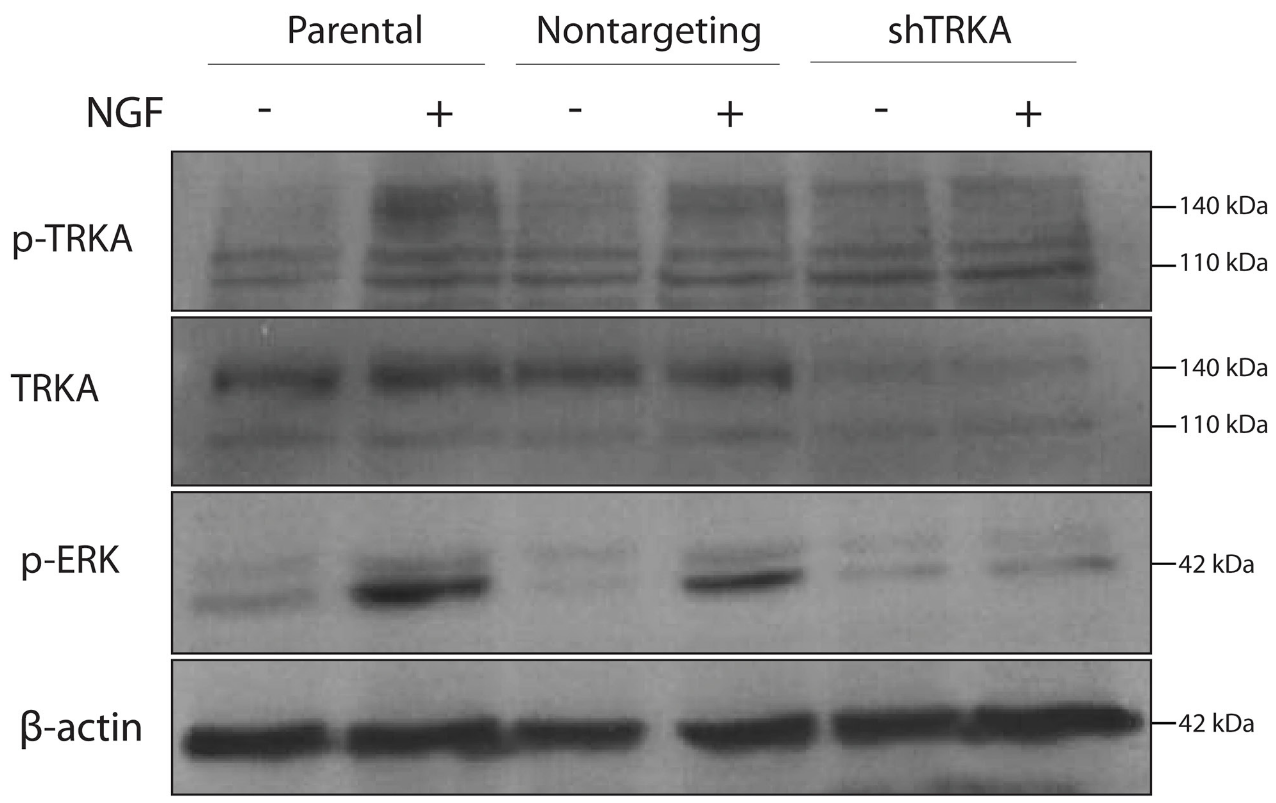


**Supplementary Figure 4: TRKA signaling in TRKA knockdown TF1 cells.** Western blot analysis of unstimulated or NGF-stimulated (100ng/mL for 15 minutes at 37°C) extracts from nontargeting shRNA, TRKA shRNA, or parental TF-1 cells using anti-p-TRKA, ant-TRKA, anti-p-ERK antibodies with a b-ACTIN loading control.

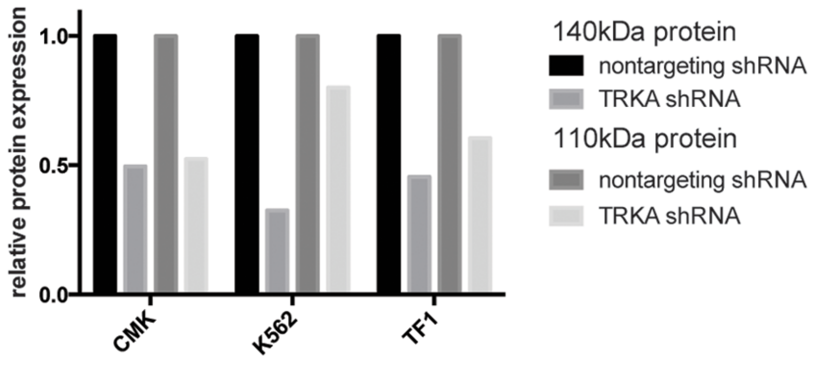


**Supplementary Figure 5: Protein knockdown by isoform.** TRKA proteins expression in shRNA knockdown and control cells was quantified per isoform. Protein expression for cells with TRKA shRNA was normalized to abundance in cells transfected with the nontargeting scrambled shRNA control.

**Supplementary Table 1: Antibodies used for CyTOF staining.**

| Isotope | Mass Channel | Marker | Clone | Source | Catalog # | Surface / Intracellular |
|---|---|---|---|---|---|---|
| 143Nd | 143 | CD117 | | DVS-Fluidigm | 3143001B | surface |
| 144Nd | 144 | CD11b | ICRF44 | DVS-Fluidigm | 3144001B | surface |
| 145Nd | 145 | CD123 | 7G3 | BD | 554527 | surface |
| 148Nd | 148 | CD34 | 581 | DVS-Fluidigm | 3148001B | surface |
| 150Nd | 150 | CD61 | VI-PL2 | DVS-Fluidigm | 3150001B | surface |
| 151Eu | 151 | CD271 | ME20.4 | BioLegend | 345102 | surface |
| 152Sm | 152 | p-STAT3 | M9C6 | Cell Signaling | 4113BF | intracellular |
| 153Eu | 153 | CD45RA | HI100 | DVS-Fluidigm | 3153001B | surface |
| 154Sm | 154 | CD45 | HI30 | DVS-Fluidigm | 3154001B | surface |
| 155Gd | 155 | p-TRKA | poly | Abcam | ab111606 | intracellular |
| 156Gd | 156 | p-MAPK | G9 | Cell Signaling | 9255BF | intracellular |
| 158Gd | 158 | CD33 | WM53 | DVS-Fluidigm | 3158001B | surface |
| 159Tb | 159 | p-AKT | M89-61 | BD | 560397 | intracellular |
| 160Gd | 160 | p-PI3K | poly | Abcam | ab61801 | intracellular |
| 161Dy | 161 | CD19 | HIB19 | BioLegend | 302202 | surface |
| 162Dy | 162 | TRKA | poly | R&D | AF175 | surface |
| 164Dy | 164 | p-mTOR | D9C2 | Cell Signaling | 5536BF | intracellular |
| 165Ho | 165 | CD16 | 3G8 | DVS-Fluidigm | 3165001B | surface |
| 166Er | 166 | CD41 | HIP8 | BioLegend | 303702 | surface |
| 167Er | 167 | p-ERK | D13.14.4E | DVS-Fluidigm | 3167005A | intracellular |
| 168Er | 168 | CD38 | HIT2 | BioLegend | 303502 | surface |
| 169Tm | 169 | p-MEK1/2 | 41G9 | Cell Signaling | 9154BF | intracellular |
| 170Er | 170 | CD3 | UCHT1 | DVS-Fluidigm | 3170001B | surface |
| 171Yb | 171 | CD90 | 5E10 | BioLegend | 328102 | surface |
| 172Yb | 172 | CD235a | HI264 | BioLegend | 349102 | surface |
| 173Yb | 173 | CD14 | HCD14 | BioLegend | 325602 | surface |
| 174Yb | 174 | p-FLT3 | 30D4 | Cell Signaling | 3464BF | intracellular |
| 175Lu | 175 | p-STAT5 | C71E5 | Cell Signaling | 9314BF | intracellular |
| 176Yb | 176 | Ki67 | 56 | BD | 556003 | intracellular |
